# Ten-year health impact, economic impact and return on investment of the South African molecular diagnostics programme for HIV, tuberculosis and SARS-CoV-2

**DOI:** 10.1136/bmjgh-2024-015830

**Published:** 2024-12-03

**Authors:** Brooke E Nichols, Alexandra de Nooy, Naseem Cassim, Lucia Hans, Manuel Pedro da Silva, Kamy Chetty, Kyra H Grantz, Alvin X Han, Andrew N Phillips, Lise Jamieson, Lesley E Scott, Wendy S Stevens

**Affiliations:** 1 Impact Department, FIND, Geneva, Switzerland; 2 Department of Global Health, Boston University School of Public Health, Boston, MA, USA; 3 Wits Diagnostic Innovation Hub, Faculty of Health Sciences, University of the Witwatersrand, Johannesburg, South Africa; 4 Department of Global Health, Amsterdam University Medical Center, Amsterdam, The Netherlands; 5 National Health Laboratory Service, Johannesburg, South Africa; 6 Department of Molecular Medicine and Haematology, Faculty of Health Sciences University of the Witwatersrand, Johannesburg, South Africa; 7 Department of Medical Microbiology, Amsterdam University Medical Center, Amsterdam, The Netherlands; 8 Institute for Global Health, University College London, London, UK; 9 Health Economics and Epidemiology Research Office, University of the Witwatersrand Faculty of Health Sciences, Johannesburg, South Africa; 10 The South African Department of Science and Innovation/National Research Foundation Centre of Excellence in Epidemiological Modelling and Analysis (SACEMA), Stellenbosch University, Stellenbosch, South Africa

**Keywords:** Tuberculosis, HIV, Health economics, Diagnostics and tools

## Abstract

**Introduction:**

To ensure there is adequate investment into diagnostics, an understanding of the magnitude of impact and return on investment is necessary. We, therefore, sought to understand the health and economic impacts of the molecular diagnostic programme in South Africa, to deepen the understanding of the broad value of diagnostics and guide future healthcare investments.

**Methods:**

We calculated the 10-year (where data were available) total cost and disability-adjusted life-years (DALYs) averted associated with molecular testing for tuberculosis diagnosis (2013–2022), HIV viral load monitoring (2013–2022), early infant diagnosis of HIV infection (2013–2022) and SARS-CoV-2 testing (2020–2022), based on the actual number of molecular tests conducted in South Africa for the respective time periods. We then calculated the economic value associated with those health gains and subsequent return on investment.

**Results:**

Since the inception of the molecular diagnostics programme in South Africa, approximately 4.3 million DALYs (uncertainty range (UR): 2.8–5.8 million) have been averted as a direct consequence of this programme. This has generated an estimated US$28.3 billion in economic value due to these health gains (UR$18.4–UR$38.7 billion). The return on investment varied by specific diagnostic test (20.3 (UR 15.2–25.4) for tuberculosis, 7.7 (UR 1.6–13.9) for HIV viral load testing, 63.0 (UR 63.0–65.5) for early infant diagnosis of HIV and 2.5 (UR 0.7–4.6) for SARS-CoV-2), for an average of 13.9 (UR 9.0–18.9) for the entire molecular diagnostics programme or US$13.9 of value for each UR$1 invested.

**Conclusions:**

The molecular diagnostics programme in South Africa generated a significant amount of health gains and economic value associated with these health gains. The return on investment rivals other high-impact public health interventions such as childhood vaccination. The molecular diagnostics programme in South Africa is highly impactful and will continue to be an excellent investment in South African public health expenditure.

**WHAT IS ALREADY KNOWN ON THIS TOPIC**

**WHAT THIS STUDY ADDS**

- This study provides a comprehensive 10-year evaluation of South Africa’s molecular diagnostics programme for TB, HIV and SARS-CoV-2, quantifying both health and economic impacts.
- This study provides some of the first estimates of returns on investment for TB, HIV and SARS-CoV-2 diagnostics, demonstrating the broad value of diagnostics across different diseases.
- The return on investment of molecular diagnostics rivals other impactful public health tools and interventions.

**HOW THIS STUDY MIGHT AFFECT RESEARCH, PRACTICE OR POLICY**

- Our study provides an evidence base to support the continued investment in the molecular diagnostic programme in South Africa.
- Diagnostics, an often-forgotten element in healthcare budgets and responses, should be priortitised as high-value interventions in both South Africa and in the global public health space.

## Introduction

 Diagnostics play a vital role in ensuring quality healthcare at an individual level—through disease diagnosis and screening as well as condition and treatment monitoring—and at the global, national or regional level through population disease surveillance.^1^ However, the importance of diagnostics is frequently overlooked, resulting in underfunded or under-resourced programmes.^2^ It is estimated that approximately 47% of the global population has limited (if any) access to diagnostics, with alarming disparity between the Global North and the Global South.^2^ Further, in comparison with other key healthcare elements, such as medicine, investment in diagnostics has historically been limited.^3^ This is concerning given the direct connection between diagnosis and treatment—and has resulted in significant empiric treatment across the disease spectrum, resulting in overtreatment, undertreatment or incorrect treatment.^3^ Additionally, although there is notable evidence of the substantial diagnostic gap in many low-income and middle-income countries—particularly for key disease areas such as HIV, tuberculosis (TB) and diabetes—there is little high-level literature which demonstrates the direct and long-term health and economic impacts of strong and well-distributed public health diagnostic programmes.^4^ This lack of evidence has limited the broad country-level investments in diagnostics needed to ensure quality healthcare for all.

In terms of diagnostic access, South Africa is a leader within the South African Development Community (SADC) region of sub-Saharan Africa. The country’s National Health Laboratory Service (NHLS) has a network of centralised molecular laboratory facilities and a daily specimen transportation and results delivery system. There is coverage at all public healthcare facilities across the country, to provide diagnostic service support to ~85% of the total population and 100% of the public sector.^5^,^7^ Further, the NHLS conducts millions of tests (both molecular and other) each year across a range of diseases, with nearly 107 million tests being performed in 2021–2022.^8^ Of these, approximately 10 million (9.4%) were molecular tests performed by the NHLS that support the long-standing HIV and TB treatment programmes and more recently the SARS-CoV-2 programme.^8 9^ These include HIV viral load testing, early infant diagnosis (EID) for HIV infection, TB diagnosis, as well as SARS-CoV-2 diagnosis. Over the last decade, molecular diagnostics have been introduced to each programme at different time points. In 2004, molecular diagnostics were introduced for viral load monitoring of people living with HIV on antiretroviral treatment. In 2011, the molecular diagnostics programme for TB was introduced, replacing the standard of care for TB diagnosis at the time—the less accurate smear microscopy. The molecular diagnosis programme expanded to include EID of HIV in 2013. These molecular platforms were then leveraged for the diagnosis of SARS-CoV-2 infection in 2020.

To ensure there is ongoing adequate and sustainable investment in diagnostics, an understanding of the magnitude of impact and return on investment (ROI) is necessary. We, therefore, investigated the 10-year health and economic impacts of the public health molecular diagnostic programme in South Africa to deepen the understanding of the broad value of diagnostics to guide future healthcare investments.

## Methods

### Study population

To determine the health and economic impact of the molecular diagnostics programme, the annual number of diagnostic tests performed between 2013 and 2022 was ascertained from the literature. Each respective diagnostic test conducted was then assigned a health impact (determined using previously published mathematical models described below for each of the different tests), and a subsequent economic impact that results from any improvement in health. The total number of diagnostic tests conducted has been consolidated and reported in online supplemental table 1.

### Health impact of a molecular diagnostic test

The health impact, in terms of disability-adjusted life years (DALYs), of each of the molecular diagnostic tools was calculated separately (table 1). The health impact for TB, HIV viral load, EID of HIV and SARS-CoV-2 molecular testing were all sourced from the literature or updated from existing models in the literature. These were used to understand both the direct clinical benefits of diagnosis and the extended benefits that diagnostic testing or monitoring associated with a potential reduction in disease transmission can have. Impact can result from multiple tests of a single individual (eg, a TB diagnosis and a viral load monitoring test), and these benefits are assumed to be additive.

**Table 1:** The cost per test and DALYs assumed per test or diagnosis as noted per disease area supported by the molecular diagnostics programme in South Africa

Test conducted	Lab price per test (2022 USD)	Total cost per test (including staff and consumables for specimen collection) (2022 USD)	Source	DALYs averted per diagnosis or per test conducted (uncertainty range)	Source
TB molecular diagnosis	US$14.01	US$17.41	NHLS,^27^	1 per diagnosis (0.75–1.25)	15 27 28
HIV viral load	US$24.00	US$28.10	NHLS,^27 28^	0.0328 per test conducted (0.007–0.059)	17 1927 28
Early infant diagnosis of HIV	US$27.73	US$32.84	NHLS,^27 28^	24.6 per diagnosis (24.6–25.6)	21 27 28
SARS-CoV-2 molecular diagnosis	US$28.63	US$32.03	NHLS,^27^	0.0745 per positive diagnosis (0.0171–0.120)	24 25 27
Sputum smear microscopy*	US$1.88	US$5.71	27	1 per diagnosis (0.75–1.25)	15 27

* Only used to calculate the counterfactual of the TB molecular diagnosis programme.

DALYs, disability-adjusted life-years; NHLS, National Health Laboratory Service; TB, tuberculosis.

#### Diagnosis of TB

The total number of TB tests conducted and the number of positive diagnoses through the molecular diagnostics programme in South Africa were sourced from the literature.^10^ We compared the total net impact of the molecular TB programme as well as the incremental impact compared with if the sputum smear microscopy (SSM) programme had continued as it was in 2010. For the counterfactual SSM programme, we assumed that 1.329 million tests per year would be conducted from 2013 to 2022 (the same number of tests conducted in 2010 when the SSM diagnostic programme was at capacity).^11^ We then assumed a constant 10% prevalence of TB among those tested, a 68% test sensitivity, and a 98% test specificity of SSM.^11^ The sensitivity and specificity of Xpert MTB/Rif^12^ were assumed to be 85% and 98%, respectively, and the sensitivity and specificity of Xpert MTB/Rif Ultra^13^ were assumed to be 91% and 99%, respectively.^14^ For the total impact of the programme, we multiplied the number of DALYs averted per correct positive TB diagnosis (assumed to be 1 DALY per diagnosis^15^) by the number of correct TB diagnoses reported over time^10^ (adjusting for test specificity). The benefits of the molecular programme are the results of the improved accuracy of molecular diagnostics compared with smear microscopy for TB diagnosis.

The number of DALYs assumed per diagnosis was derived from an analysis of TB testing across three different countries (South Africa, China and India) over a 10-year time horizon.^15^ For all three countries, despite high variation in TB incidence (from 75/100 000 in China to 993/100 000 in South Africa) and in HIV prevalence (from 0.058% in China to 17.3% in South Africa), 1 DALY was averted per one additional person diagnosed through active case finding. For these estimates, differing assumptions regarding TB were made based on HIV status (including transmissibility, risk of reinfection when latently infected, rate of stabilisation, rate of treatment duration, etc). Full-analysis parameters have been reported in tables1  2 of Azman *et al*.^15^ Despite small variations in estimates between countries, we have reported on an uncertainty range (UR) (0.75–1.25 DALYs averted per diagnosis) evaluated in a follow-up manuscript reported in online supplemental table S1 by Brümmer *et al*.^16^

**Table 2:** The return on investment of the molecular diagnostics programme in South Africa, as expressed by the total economic value of the health returns divided by the costs of conducting those tests across all years considered in this analysis

Test conducted	Economic value of health gains (2022 USD) (uncertainty range)	Total cost of implementation (2022 USD)	Return on investment (uncertainty range)
TB molecular diagnosis*	US$6.4 billion (US$4.8–US$8.0 billion)	US$315 million†	20.3 (15.2–25.4)
HIV viral load	US$11.9 billion (US$2.1–US$17.7 billion)	US$1.27 billion	7.7 (1.6–13.9)
Early infant diagnosis of HIV	US$11.3 billion (US$11.3–US$11.8 billion)	US$179 million†	63.0 (63.0–65.5)
SARS-CoV-2 molecular diagnosis	US$0.8 billion (US$0.2–US$1.3 billion)	US$273 million	2.8 (0.7–4.6)
Total	US$28.3 billion (US$18.4–US$38.7 billion)	US$2.0 billion	13.9 (9.0–18.9)

* Incremental benefit compared with a counterfactual of a smear microscopy programme.

† Including healthcare costs associated with false positive test results.

TB, tuberculosis.

#### HIV viral load monitoring

The HIV synthesis model, an individual-based stochastic simulation model that includes HIV transmission, progression, HIV-related treatment and prevention interventions, was used to estimate the DALYs associated with a viral load test.^17 18^ Two scenarios were run to tease out the incremental benefit of viral load testing: (1) no viral load testing for 3 years, followed by fully implemented viral load testing and (2) fully implemented viral load testing over 10 years. The difference in DALYs between the two scenarios provides an indication of the DALYs averted because of viral load testing alone. Through sampling of parameter values at the start of each model run, we create 300 ‘setting scenarios’ reflecting uncertainty in assumptions and a range of characteristics. Parameters include HIV prevalence in individuals aged 15–49: 11% (range 1%–32%), of those with HIV, the proportion diagnosed: 91% (range 84%–97%), of those diagnosed, the proportion on ART: 95% (range 89%–98%); of those on ART, the proportion with viral suppression <100 copies/mL 93% (range 83%–99%).

Effects of viral load monitoring have been previously described (viral load informed differentiated care^19^). Measurement of a viral load above 1000 copies/mL prompts enhanced adherence counselling which has a probability of leading to an improvement in adherence. Also, switches in regimen can only be made if the viral load has been confirmed to be above 1000 copies/mL after enhanced adherence counselling. We calculated the mean difference in the number of HIV- related deaths and total DALYs between the two scenarios during the 3-year period. Taking the global burden of disease DALY approach (adding the years of life lost in the period that a death has occurred),^20^ we estimated a mean of 6797 (UR of ±2 SEs of the mean DALYs averted: 1481–12 113) DALYs averted per 3 months, in the context of an adult population of 10 million with 207 000 viral load tests done per 3 months compared with when no viral load tests were done. This resulted in 0.0328 (UR 0.007–0.059) DALYs averted for each viral load conducted.

We then multiplied the annual number of viral load tests conducted by the NHLS from 2013 to 2022 by the number of DALYs averted per viral load conducted. Given that we calculated the impact per viral load test conducted (rather than per elevated viral load result), specific test accuracy parameters were not taken into account.

#### HIV EID

A model of quality assurance systems for HIV EID estimated the DALYs associated with false negative test results with and without these systems for five countries.^21^ This study indicated that the DALYs associated with an undiagnosed case of HIV among infants was 24.6 in South Africa (derived in the manuscript by dividing the total number of DALYs associated with missed HIV+infant cases by the total number of missed HIV+infants in South Africa). The estimate of DALYs per HIV+infant missed was relatively similar across each country, and robust to the wide variation in EID testing coverage (from 23% in Senegal to 87% in South Africa) and perinatal HIV transmission rate among mothers living with HIV (from 4% in South Africa to 20% in Senegal). To account for potential uncertainty, we also assessed the range of DALYs associated with a missed HIV+infant case identified across all five countries (24.6–25.6).

We then multiplied the number of DALYs averted by the number of infants correctly diagnosed with HIV by year in South Africa^22^ (adjusting for test specificity). The sensitivity of HIV EID is assumed to be 99.3% and specificity 99.5%,^23^ and a second test is required for confirmation.

#### Diagnosis of SARS-CoV-2

We used results from the Propelling Action for Testing and Treating simulation model of SARS-CoV-2 transmission to estimate the DALYs averted per SARS-CoV-2 diagnostic test used in countries most similar to South Africa in terms of population demography and household size.^24 25^ The impact of testing was primarily due to the reduction in the number of onward infections following isolation of positive cases, rather than any specific clinical impact. Individuals were assumed to reduce their number of community contacts by 50% on average after receiving a positive diagnosis but were not assumed to be able to effectively isolate from household members. Given that the relative impact of testing for SARS-CoV-2 is dependent on several parameters, we took the median and IQR of impact across effective reproductive numbers of SARS-CoV-2 from 1.2 to 2.0, a testing rate of 100 tests per 100 000 per year, and minimal vaccine coverage (10% coverage). (Additional testing and transmission parameters can be found in Han *et al* in table 1).^24^ This resulted in 0.0745 (UR 0.0171–0.120) DALYs averted per correct positive test result (adjusted for test specificity). The sensitivity of molecular SARS-CoV-2 testing was assumed to be 99% and specificity 97%.^26^

### Costs of diagnostic tests

The cost of each individual diagnostic test was derived from a combination of the NHLS-reported test prices for each test. This test cost is the amount reimbursed to the NHLS by the national government but does not include the costs related to the healthcare visit required to take the sample from the person seeking care. The final cost per test included these healthcare visit-related costs derived from South Africa HIV and TB costing data.^27 28^ All assumptions and cost data included can be found in online supplemental table 2.

### Economic impact of health gains and ROI

The total number of DALYs per health area was calculated per year and multiplied by the GDP per capita per respective year to calculate the economic impact of the molecular diagnostics programme. Results are reported for each diagnostic test and for the entire programme (table 1). No DALYs are averted for false positive results at the end of a diagnostic algorithm, calculated using the average expected incidence of infection for each health area and the specificity of the test or test algorithm. All health benefits and related economic gains are expressed in terms of the net present value of these gains. The health benefits (DALYs averted) of a TB molecular diagnostic are expected to occur over a lifetime after the average age of active TB disease, the benefits of HIV viral load testing were calculated over a 10-year period (with full life years lost added to the year in which any death has occurred) (discounted at 3% annually), EID over a lifetime (discounted at 3% annually) and SARS-CoV-2 testing over 1 year (as such, not discounted).

ROI was calculated as the total economic impact per diagnostic tool divided by the total cost of all diagnostic tests required to generate that impact (including both ‘positive’ and ‘negative’ tests). The total number of tests per programme, across the respective time periods considered, was taken from the literature for TB, HIV viral load, SARS-CoV-2 and EID.^1022 29^,^31^ To calculate the number of HIV EID tests conducted on average for 2020–2022, we used the number of live births to women living with HIV in 2021 (~275 527), the EID testing algorithm in South Africa and the coverage of testing at each part of the algorithm (averaged between the National Institute for Communicable Diseases of South Africa (NICD) and District Health Information Software (DHIS) sources). Given the relative overcounting of infants diagnosed with HIV reported in the literature,^22^ we also adjusted the numbers of positive infants reported in 2013–2019 down to reflect this overcounting. Additionally, we adjusted for the neonatal mortality rate in South Africa for a number of infants expected to be tested at 10 weeks, and the number of infants testing positive who would not need to be retested.^32^

The total costs were calculated as the 2022 test price per test in South African Rands (ZAR), converted to US dollar (USD) using the average 2022 conversion rate (ZAR16.37 to US$1^33^), and multiplying each respective test cost by the total number of tests of that type conducted per year since the inception of the molecular diagnostic programme. ‘Deadweight’ costs have been removed from the ROI analysis for TB diagnosis by using the appropriate incremental gains of the molecular programme (eg, removing the costs associated with the counterfactual of keeping the SSM programme running at the same scale as when the molecular programme had been introduced). The additional costs incurred associated with false positive test results for TB and EID were calculated using the prevalence of infection and test specificity of the testing algorithm. These additional costs included those for unnecessary treatment of drug-sensitive TB disease, US$112.70,^27^ and unnecessary lifetime ART treatment cost for someone misdiagnosed with HIV, US$6115 (discounted at 3% per year, assuming a life expectancy of 63.8 years).^21^

### Uncertainty in estimates

The number of tests conducted and the cost per test conducted do not have inherent uncertainty. The UR in our estimates is derived from the estimated health benefit of a diagnostic test or a positive diagnosis. Across all health areas, we assumed an UR in the health benefit (DALYs) that were considered in the originating publications and have been described in the DALY estimates above. Though each UR was calculated in differing ways depending on how the modelling results were reported (described above), they are reported throughout the manuscript uniformly as a UR. This UR then results in uncertainty for not only the DALY calculations but also onward economic and ROI calculations.

### Ethics and patient/public involvement

Aggregate data on the number of molecular tests conducted was obtained directly from previously published manuscripts.^10 22 29 30^ Any missing data points were imputed rather than extracted from raw data. The annual number of molecular diagnostics by health area is reported in online supplemental table 1. Given the lack of primary data collection for this analysis, there was no patient or public involvement in the development of this manuscript.

## Results

### Health impact

**Figure 1:**
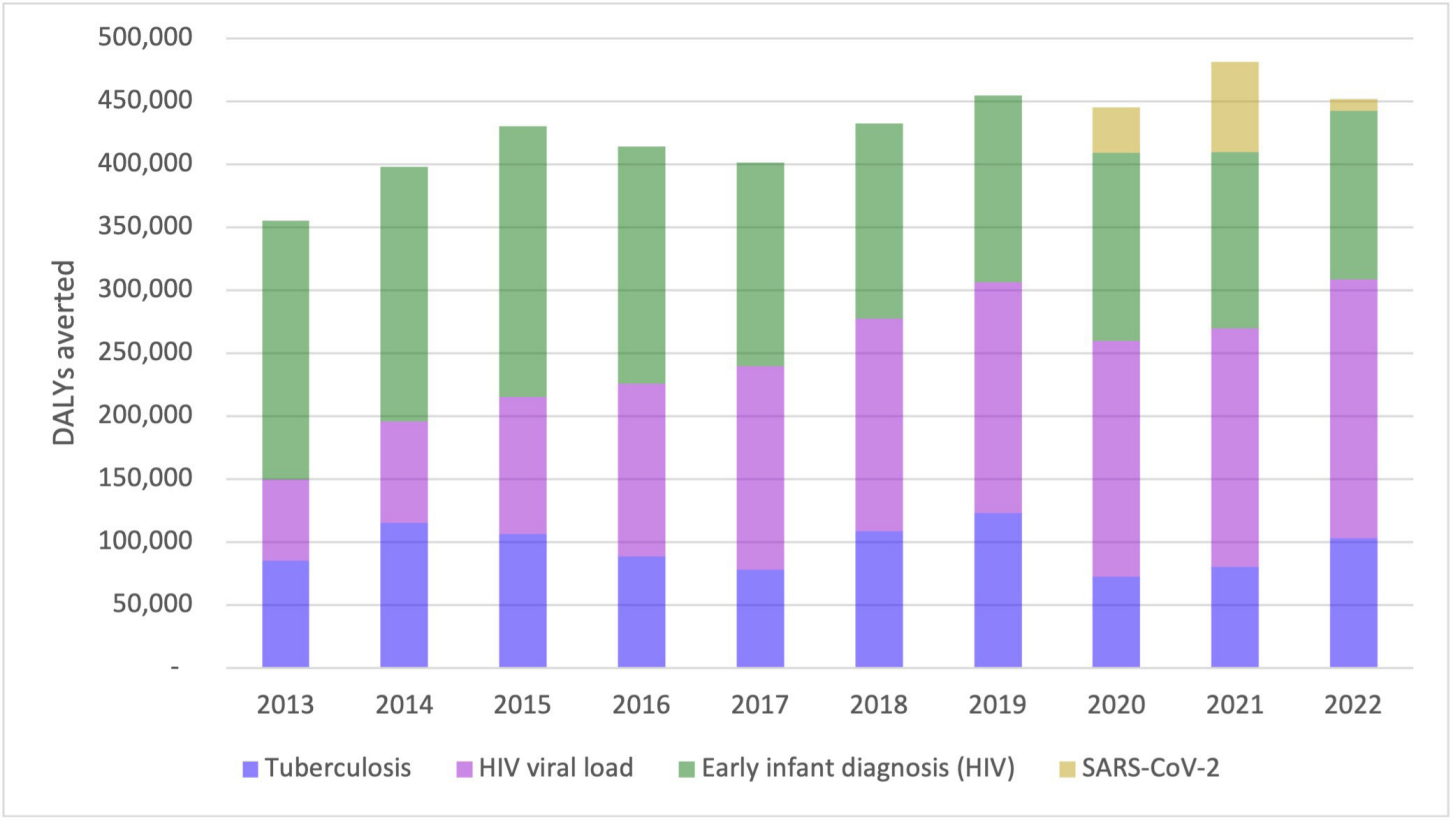
The average annual DALYs averted of South Africa’s public sector molecular diagnostics programme for HIV, TB and SARS-CoV-2 from 2013 to 2022. DALY, disability-adjusted life-year; TB, tuberculosis.

Since the inception of the molecular diagnostics programme in South Africa, 4.3 million DALYs (UR 2.8–5.8 million) have been averted as a direct consequence of this programme. This includes 1.0 million incremental DALYs averted due to the diagnosis of TB (2013–2022) (incremental to a continued smear microscopy programme, adjusting for false positive test results; UR 0.7–1.2 million), 1.5 million averted due to HIV viral load tests (2013–2022) (UR 0.3–2.7 million), 1.7 million due to EID (2013–2022) (UR 1.7–1.8 million) and 120 000 DALYs averted due to molecular SARS-CoV-2 testing (2020–2022) (UR 30 000–190 000). Figure 1 highlights the relative impact per year that each of the diagnostics has demonstrated.

### Economic impact of health gains

**Figure 2:**
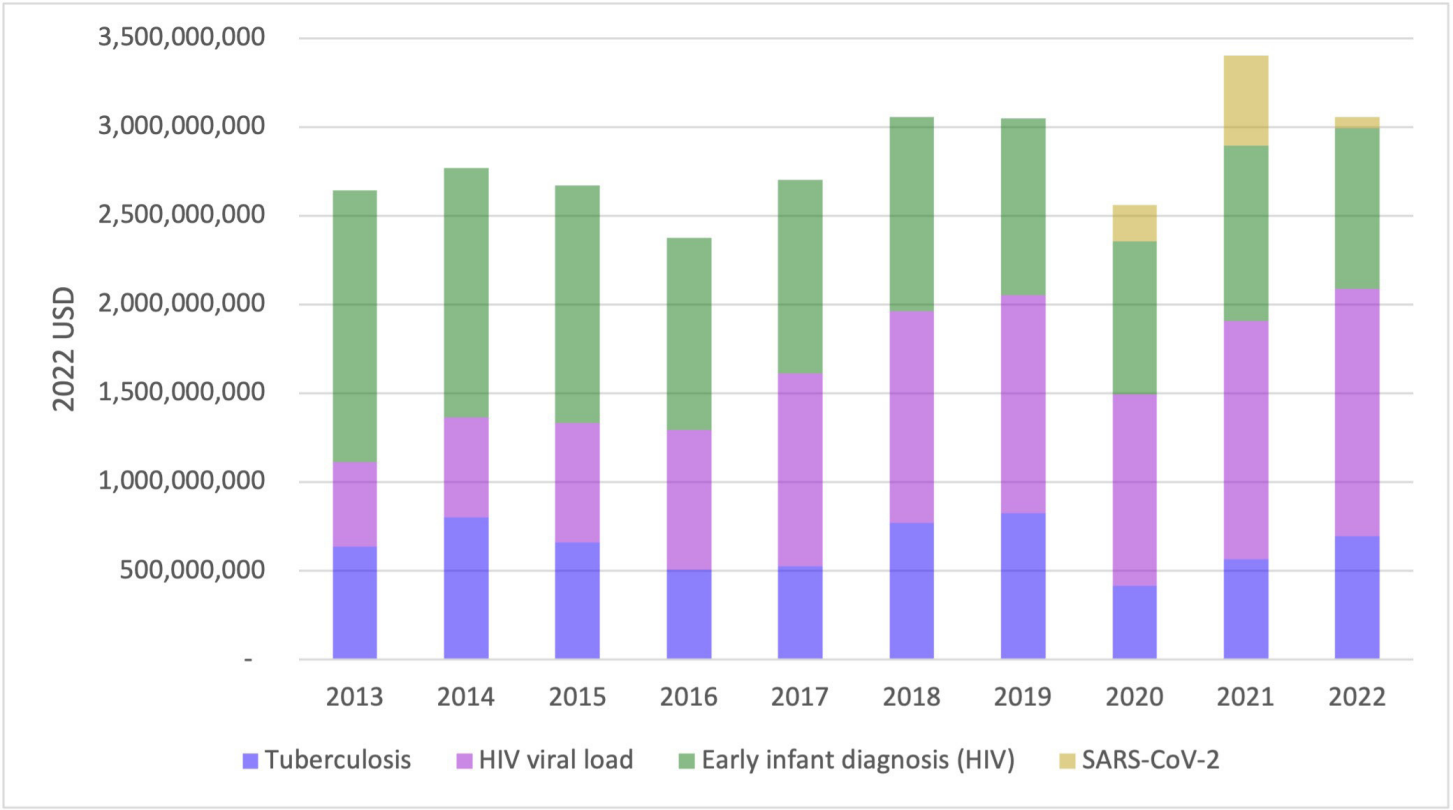
Annual economic value (in 2022 USD) associated with the health gains of the molecular diagnostics programme for HIV, TB and SARS-CoV-2 from 2013 to 2022. TB, tuberculosis.

The total economic value of the molecular diagnostics programme due to health gains over the 10-year period was US$28.3 billion (figure 2) (UR US$18.4–US$38.7 billion). This includes US$6.4 billion in economic value associated with improved health related to TB diagnosis (2013–2022) (UR US$4.8–US$8.0 billion), US$11.9 billion for HIV viral load testing (2013–2022) (UR US$2.1–US$17.7 billion), US$11.3 billion for HIV EID (2013–2022) (UR US$11.3–US$11.8 billion) and US$0.8 billion for SARS-CoV-2 testing (2020–2022) (UR US$0.2–US$1.3 billion).

### Return on investment

The molecular TB diagnostics programme has cost an estimated US$418 million over 10 years (for all TB diagnostics conducted plus costs associated with false positive test results), while our counterfactual was assumed to cost US$103 million over the same period (for diagnostics plus costs associated with false positive results), for an incremental cost of US$315 million of the molecular TB diagnostics programme. The total incremental economic value of health gains associated with TB molecular diagnosis, divided by the total incremental cost of the molecular TB diagnostics programme resulted in an ROI of 20.3 or US$20.3 of economic value for each US$1 invested in diagnosis (UR 15.2–25.4). The ROI for HIV viral load was 7.7 (UR 1.6–13.9), for HIV EID was 63.0 (UR 63.0–65.5), and for SARS-CoV-2 diagnosis was 2.8 (UR 0.7–4.6). Averaged across the whole molecular diagnostics programme, the average ROI was 13.9 or US$13.9 of economic gain related to improved health for each US$1 invested in diagnostics (UR 9.0–18.9).

## Discussion

Generally, molecular testing has multiple roles in disease management, including diagnosis, monitoring response to treatment and guiding decisions on appropriate management (eg, indications for HIV drug resistance tests).^9^ Further, as compared with other diagnostic counterparts (like TB culture), molecular tests are frequently able to return accurate results more rapidly, and hence allow for action to be taken at an earlier stage.^9^ Thus, molecular testing, if well implemented, has the potential to greatly improve patient outcomes. This has been shown by the public sector molecular diagnostics programme in South Africa that generated a significant amount of health gains and economic value associated with these health gains. The ROI was ≥2.8 for all individual tests, as high as 63.0 and 13.9 on average—suggesting excellent value for money across all molecular tests. These levels of ROIs for some tests rival childhood vaccination programmes (ROI 16)—often considered one of the most valuable public health interventions.^34 35^ While the ROI value for each of the individual types of molecular tests varied greatly, each still resulted in a positive impact on the South African healthcare system given that all tests had, on average, at least a full ROI.

The only test in which the UR spanned to below an ROI of 1 was for SARS-CoV-2. This is due to the dynamic impact of SARS-CoV-2 testing that strongly depends on the time point in an epidemic wave that people are testing in and the effective reproductive number of the variant of consideration. This highlights that for pathogens for which the UR dips below 1 ROI for a molecular diagnostic, the use of a rapid diagnostic test in place of a molecular diagnostic test may ensure a positive ROI due to the substantially lower cost per test.

While the total amount of molecular diagnostics being conducted from pre to during the pandemic remained relatively stable, there was a clear reduction in the amount of TB testing done and a substantial amount of SARS-CoV-2 testing conducted. This reduction in TB testing has been reported elsewhere.^36 37^ Importantly, the reduction in TB testing was not due to demand for SARS-CoV-2 testing, but rather because of healthcare seeking behaviour in general. If the COVID-19 pandemic had not taken place, there would not have likely been the same decrease in TB testing over the 2020–2022 period. Given the relatively greater health impact of TB diagnostics, this would have resulted in a greater overall health and economic impact of the molecular diagnostics programme over the 2013–2022 period. This would not, however, have affected our overall calculations on ROI is based on the constant incremental per-test impact and costs.

Comparing the ROI of molecular diagnostics to other public health tools such as vaccines and therapeutics, for each specific disease area, is useful to understand the relative role of diagnostics as part of a public health programme. For SARS-CoV-2, the ROI of vaccination has not been estimated for South Africa, but in New York City was estimated to be 10 to 1.^38^ If adjusting this estimate for the GDP per capita in South Africa and the smaller proportion of people at risk for severe disease and death in South Africa (due to demographic differences), the ROI for SARS-CoV-2 vaccines in South Africa might approach the value of diagnostics in South Africa. ROIs for specific therapeutics for SARS-CoV-2 in South Africa have not been calculated as therapeutics such as paxlovid, have not been available in South Africa. For both TB and HIV, the ROI for investments into vaccines has been effectively 0 (or negative) given that there are no vaccines available for HIV, and no effective vaccines for adults for TB (with the BCG vaccine for TB protective for <5-year-old only). For HIV, the UNAIDS ‘Fast-Track’ strategy which focuses on investments in expanding HIV service coverage (as opposed to maintaining constant coverage) estimates an approximate ROI of 6.46 to 1 for Southern Africa.^39 40^ This contextualises that viral load testing is complementary to a broader package of HIV-related services, with an ROI of 2, and that EID remains to have an extraordinarily high ROI. With respect to TB, there also exists a set of services which together have resulted in an estimated drop of more than 50% in the incidence rate between 2011 and 2022.^41^ While it is difficult to quantify exactly how much each particular service (eg, the increase in TB diagnostic capacity and accessibility, increased TB treatment options and the impact of improved HIV services) has contributed towards reduced incidence, the introduction of Xpert MTB/Rif and Ultra and the scale-up of the molecular testing programme has had a clear impact. Similarly in terms of investment, while there is no specific literature available on the ROI of TB diagnostics, studies have shown that TB prevention and care yields an ROI of 43—implicitly including diagnosis—whereas we have estimated the ROI of diagnosis aspect of prevention and care to be 20.3.^42^

These values represent one consistent methodological way to calculate impact, economic value and ROI. There are several changes in the underlying assumptions that might influence the direction of these values in both directions. First, the average GDP per capita in South Africa may not reflect the burden of disease where the health gains occurred for each respective disease (eg, slightly lower burden of disease among higher household social economic index score for TB infection).^43^ If including these differences, the economic gains might be slightly overstated where the benefits occurred among individuals with lower annual GDP. We did not, however, want to weight the economic value of health differently among the South African population. In the other direction, within the scope of this analysis, we have only calculated the economic value of the health gains associated with molecular diagnostics rather than the total economic benefit. The total economic benefit likely far exceeds what we have estimated here—for example, the benefits associated with being able to return to work after a negative SARS-CoV-2 test during the pandemic or benefits associated with the reduced frequency of healthcare clinic visits required after a suppressed viral load test—reducing costs to individuals on antiretroviral treatment and possibly increasing economic activity due to fewer days spent seeking care. Estimating the total value was outside the scope of this analysis.

There were several limitations to our analysis. First, we did not include the value related to drug susceptibility testing for TB given the paucity of literature on the DALYs associated with TB resistance testing. If we had included that, the likely value and impact of the molecular diagnostic programme would have only increased, especially since the diagnosis of rifampicin resistant TB is provided simultaneously with the molecular diagnosis of TB. Second, for the incremental impact of the TB molecular diagnostics programme, we assumed that the size of the smear microscopy programme would have held constant from 2010 onwards. An expansion of this programme would have generated more health effects and related economic value of health gains, resulting in a diminished incremental benefit of the molecular programme. We made this assumption, however, given that the microscopy programme was already at capacity in 2010. Third, for the HIV viral load DALY calculations, it was assumed that individuals would be on a dolutegravir-based first-line antiretroviral treatment regimen. The DALYs per viral load would have likely been greater in predolutegravir era, given that previous regimens were slightly less effective. This would have resulted in a slight underestimate of the benefit of viral load testing before the introduction of dolutegravir-based first-line antiretroviral treatment regimens—but do reflect the likely benefit moving forward. Fourth, while we have presented here the net present benefits of the molecular diagnostics programme, the time horizon of the economic benefit associated with health gains differs between diseases. Finally, we did not consider the impact of potential additional costs incurred as a result of test sensitivity given the uncertainty around the costs that may occur as a consequence (eg, false negative tests). Individuals who receive a false negative result may need to seek care again (or multiple times) to receive a correct diagnosis, incurring additional costs (most likely with TB testing, less so for SARS-CoV-2 given the short duration of infection). These individuals may also transmit infection if they are diagnosed negative (for TB, SARS-CoV-2, possibly for viral load if individuals think they are suppressed; not likely for EID). It is unlikely, however, that these tests will influence our results significantly, and the test sensitivity of molecular diagnostics is relatively high compared with other available diagnostics.

## Conclusions

In conclusion, the molecular diagnostics programme in South Africa is highly impactful and will continue to be an excellent investment in South African health expenditure. The value and ROI of these diagnostic tools are in line with other highly impactful public health interventions and should continue to be valued as such. Into the future, molecular diagnostics are likely to play a more significant role in the monitoring and control of existing diseases (other than those described here) and, as seen for SARS-CoV-2, maybe the first point of call for the diagnosis of and surveillance of future pandemics given their relative ease of development and implementation into existing systems.

## Supplementary material

10.1136/bmjgh-2024-015830 online supplemental file 1

## Data Availability

Data are available in a public, open access repository.
