# Supplementary material for: Ten-year health impact, economic impact and return on investment of the South African molecular diagnostics programme for HIV, tuberculosis and SARS-CoV-2
Source: BMJ Glob Health. 2024 Dec 3;9(12):e015830. doi: 10.1136/bmjgh-2024-015830 (PMC11624778; doi:10.1136/bmjgh-2024-015830)
Supplement: online supplemental file 1 [file bmjgh-9-12-s001.pdf]

Supplemental Table 1. Total number of molecular tests conducted the National Health Laboratory Service between 2013-2022, and positive test results (where relevant) by health area

	**Tuberculosis**		**HIV viral load**	**HIV early infant diagnosis**		**SARS-CoV-2**	
	Total molecular TB tests conducted	Total tests MTB detected	Viral loads conducted*	EID tests conducted	Positive diagnoses	Molecular SARS-CoV-2 test conducted	Positive SARS-CoV-2 tests
2013	1786860	208644	1961720	346338	8371		
2014	2384708	249708	2456139	373318	8217		
2015	2643125	245393	3316736	485647	8765		
2016	2416476	223453	4177562	565361	7682		
2017	2195762	208913	4919845	592147	6600		
2018	2165112	219159	5146126	603669	6319		
2019	2176817	233500	5587573	614506	6054		
2020	1687539	178460	5706052	612752**	6098	2766318	478296
2021	2034734	189169	5788673	618281**	5688	4482250	964580
2022	2530668	216548	6274438	623812***	5430***	1273798	125214
Source	(1)	(1)	(2)	(3)	(3,4)	(5)	(5)

*Elevated viral load tests were not explicitly considered in the analysis, and thus not reported here (the DALY averted was calculated using the total number of viral loads conducted, rather than the total number of elevated viral load results)
**Back calculated the total number of tests based on reported positivity rate and the number of reported positive diagoses.
***A linear projection of historical data (2013-2021) to estimate the missing number of tests and positive diagnoses for 2022.

Supplemental Table 2. Cost per test assumed (reported in 2022 USD), broken down by cost component, by health area

	NHLS test price*	Consumables for specimen collection provided on site**	Staff time*** (specimen collection)	Total	Source
TB molecular diagnosis	14.01	1.011	2.39	17.41	NHLS, (6)
HIV viral load	24.00	1.71	2.39	28.10	NHLS, (7)
Early infant diagnosis	27.73	1.72	3.39	32.84	NHLS, (7)
SARS-CoV-2 molecular diagnosis***	24.56	1.011	2.39	27.96	NHLS, (6)
Sputum smear microscopy	1.88	0.44	3.39	5.71	NHLS, (6)

*inclusive of transporting, testing in the centralized lab [instruments, reagents, laboratory staff], and result delivery back to health care facility. Total cost reported by NHLS & charged to the National Department of Health.

**Including gloves, masks, disinfectants, needles, specimen collection, as applicable)

***Clinic staff time for specimen collection; time assumed to be the same for SARS-CoV-2 as for TB molecular testing.

REFERENCES

1. da Silva MP, Cassim N, Ndlovu S, Marokane PS, Radebe M, Shapiro A, et al. More Than a Decade of GeneXpert®Mycobacterium tuberculosis/Rifampicin (Ultra) Testing in South Africa: Laboratory Insights from Twenty-Three Million Tests. Diagnostics (Basel) [Internet]. 2023 Oct 1 [cited 2024 Feb 9];13(20). Available from: https://pubmed.ncbi.nlm.nih.gov/37892074/
2. Hans L, Cassim N, Sarang S, Hardie D, Ndlovu S, Venter WDF, et al. HIV Viral Load Testing in the South African Public Health Setting in the Context of Evolving ART Guidelines and Advances in Technology, 2013–2022. Diagnostics 2023, Vol 13, Page 2731 [Internet]. 2023 Aug 22 [cited 2024 Feb 9];13(17):2731. Available from: https://www.mdpi.com/2075-4418/13/17/2731/htm
3. Radebe L, Haeri Mazanderani A, Sherman GG. Indeterminate HIV PCR results within South Africa’s early infant diagnosis programme, 2010–2019. Clinical Microbiology and Infection. 2022 Apr 1;28(4):609.e7-609.e13.
4. Haeri Mazanderani AF, Murray TY, Johnson LF, Ntloana M, Silere-Maqetseba T, Guo S, et al. Eliminating Vertical Transmission of HIV in South Africa: Establishing a Baseline for the Global Alliance to End AIDS in Children. Diagnostics 2023, Vol 13, Page 2563 [Internet]. 2023 Aug 1 [cited 2024 Feb 9];13(15):2563. Available from: https://www.mdpi.com/2075-4418/13/15/2563/htm
5. Scott LE, Hsiao NY, Dor G, Hans L, Marokane P, da Silva MP, et al. How South Africa Used National Cycle Threshold (Ct) Values to Continuously Monitor SARS-CoV-2 Laboratory Test Quality. Diagnostics 2023, Vol 13, Page 2554 [Internet]. 2023 Aug 1 [cited 2024 Feb 9];13(15):2554. Available from: https://www.mdpi.com/2075-4418/13/15/2554/htm
6. Coetzee L, Hirasen K, Jamieson L, Evans D, Meyer-Rath G. Linganisa TB Cost Model, South Africa. Johannesburg: Health Economics and Epidemiology Research Office, University of the Witwatersrand; 2022.

7. Meyer-Rath G, van Rensburg C, Chiu C, Leuner R, Jamieson L, Cohen S. The per-patient costs of HIV services in South Africa: Systematic review and application in the South African HIV Investment Case. PLoS One [Internet]. 2019 Feb 1 [cited 2024 Feb 23];14(2):e0210497. Available from: https://journals.plos.org/plosone/article?id=10.1371/journal.pone.0210497
